# Association between Phosphorylated AMP-Activated Protein Kinase and Acetyl-CoA Carboxylase Expression and Outcome in Patients with Squamous Cell Carcinoma of the Head and Neck

**DOI:** 10.1371/journal.pone.0096183

**Published:** 2014-04-25

**Authors:** Ying-Wen Su, Yun-Ho Lin, Man-Hui Pai, An-Chi Lo, Yu-Chieh Lee, I-Chih Fang, Johnson Lin, Ruey-Kuen Hsieh, Yi-Fang Chang, Chi-Long Chen

**Affiliations:** 1 Division of Hematology and Medical Oncology, Department of Internal Medicine, Mackay Memorial Hospital, Taipei, Taiwan; 2 Division of Oral Pathology, Department of Dentistry, Taipei Medical University Hospital, Taipei, Taiwan; 3 Department of Anatomy, Taipei Medical University, Taipei, Taiwan; 4 Good Clinical Research Center, Mackay Memorial Hospital, Taipei, Taiwan; 5 Graduate Institute of Medical Sciences, Taipei Medical University, Taipei, Taiwan; 6 Department of Pathology, Wan Fang Hospital, Taipei Medical University, Taipei, Taiwan; University of Nebraska Medical Center, United States of America

## Abstract

**Background:**

Epidemiological studies have indicated that impaired glucose metabolism may increase the risk of squamous cell carcinoma of the head and neck (SCCHN). AMP-activated protein kinase (AMPK) regulates glucose and lipid metabolism via the phosphorylation and subsequent inactivation of its downstream target acetyl-CoA carboxylase (ACC).Thus, we analyzed the expression of pAMPK and its downstream target phosphorylated acetyl-CoA carboxylase (pACC), as well as their impact on the survival of patients with resected SCCHN.

**Methods:**

One hundred eighteen patients with surgically resected SCCHN were enrolled. Immunohistochemical (IHC) staining for pAMPK and pACC was performed using tissue microarrays of operative specimens of SCCHN. The expression was divided into two or three groups according to the IHC score [pAMPK: negative (0), positive (1–3); pACC: negative (0), low expression (1, 2), and high expression (3)]. Statistical analysis was performed to determine the association of pAMPK expression with clinicopathological features and pACC and pErk expression.

**Results:**

The positive rates of pAMPK and pACC expression were 64.4% (76/118) and 68.6% (81/118), respectively. pAMPK was significantly higher in patients aged younger than 60 years (*P = *0.024; χ^2^test) and those with early-stage (T1/T2; *P* = 0.02; χ^2^ test) and oral cavity (*P* = 0.026; Fisher’s exact test) tumors. In multivariate analysis, pAMPK expression was not significantly correlated with overall survival (OS) (adjusted hazard ratio [HR]: 0.66; 95% confidence interval [CI]: 0.35–1.23), whereas high pACC expression was independently associated with worse OS in node-positive patients (adjusted HR: 17.58; 95% CI: 3.50–88.18).

**Conclusions:**

Strong expression of pACC was found to be an independent prognostic marker for patients with node-positive SCCHN. Our results suggest that pACC may play a role in tumor progression of SCCHN and may help to identify patient subgroups at high risk for poor disease outcome.

## Introduction

Squamous cell carcinoma of the head and neck (SCCHN) is the seventh leading cause of cancer death worldwide [1]. Although smoking, alcohol consumption, betel-quid chewing, and human papillomavirus infection are established risk factors for SCCHN [2], abnormalities of glucose metabolism may also play a role in SCCHN carcinogenesis. Several studies have shown that diabetes is associated with a higher prevalence of premalignant lesions such as erythroplakia and leukoplakia that predispose patients to oral cancer and an increased incidence of SCCHN [3]–[6].

AMP-activated protein kinase (AMPK) is an evolutionarily conserved serine/threonine protein kinase that has recently been shown to be a key regulator of glucose metabolism. AMPK is activated in response to metabolic stresses, such as hypoxia and ischemia, and the anti-diabetic drug metformin [7], [8]. Phosphorylation and activation of AMPK stimulates fatty acid oxidation through the phosphorylation and subsequent inhibition of its downstream target acetyl-CoA carboxylase (ACC). This leads to ATP generation and inhibition of ATP-consuming events, such as fatty acid synthesis [9]. Thus, AMPK acts as a cellular fuel sensor by controlling intracellular energy levels to maintain appropriate cell growth rates.

Several studies have indicated that AMPK exerts a protective effect against various cancers. AMPK activation has been shown to inhibit cell proliferation *in?vitro* and *in?vivo* in lung, breast, and ovarian cancers [10]–[12]. Metformin, an AMPK activator, prevented the development of oral squamous cell carcinomas from carcinogen-induced premalignant lesions in a mouse model [13]. Thus, the aim of our study was to evaluate phosphorylated AMPK (pAMPK) expression and its association with clinicopathological parameters and survival in SCCHN. The expression of phosphorylated ACC (pACC) and its association with patient survival was also determined.

## Materials and Methods

### Ethics Statement

The study protocol was approved by the institutional review board at Taipei Medical University Wan Fang Hospital (approval number: 99049). Written informed consent was obtained from all participant.

### Study Subjects and Tissue Microarray (TMA) Construction

Patients diagnosed with SCCHN of the oral cavity, oropharynx, hypopharynx, or larynx who had available staging information and received surgery and complete treatment at Taipei Medical University Wan Fang Hospital between August 1998 and July 2010 were included in the study (*n* = 134). Patients who died within 2 months after surgery (*n* = 2), those who were lost to follow up (*n* = 1), and those with insufficient samples for TMA analysis were excluded (*n* = 13). Therefore, 118 patients were included in the analysis. Formalin-fixed, paraffin-embedded (FFPE) surgical tumor samples were retrieved from the Department of Pathology at Wan Fan Hospital. FFPE tumor and paired normal mucosal tissues (3-µm thick) were arrayed in quadruplicate for TMA. Histopathological differentiation was evaluated independently by 2 pathologists (Y-H. L. and C-L. C.) according to the 2004 World Health Organization classification system. TNM (tumor–node–metastasis) staging was evaluated according to the guidelines of the American Joint Committee on Cancer [14]. A chart review was conducted to retrieve clinical and pathological information, including demographic data, TNM stage, and overall survival (OS). Patients were monitored until death or April 1, 2012. The baseline characteristics of the patients are listed in Table 1.

**Table 1:** Patient characteristics.

	Total *n = *118 (%)
**Age, median (range)**	55.0 (30.2–88.9)
<60 years	80 (67.8)
≥60 years	38 (32.2)
**Gender**	
Female	9 (7.6)
Male	109 (92.4)
**(Neo)Adjuvant**	
None	57 (48.3)
Neoadjuvant treatment only	4 (3.4)
Adjuvant treatment only	45 (38.1)
Both	12 (10.2)
**T**	
1	48 (40.7)
2	35 (29.7)
3	7 (5.9)
4	28 (23.7)
**N**	
0	81 (68.6)
1	14 (11.9)
2	23 (19.5)
**Stage**	
1	41 (34.8)
2	22 (18.6)
3	12 (10.2)
4	43 (36.4)
**Site**	
Non-oral cavity	12 (10.2)
Oral cavity	106 (89.8)

T, tumor; N, node; *n*, patient number.

### Immunohistochemistry

Immunohistochemical (IHC) staining was performed using the BenchMark ULTRA slide staining system (Ventana Medical Systems, USA). Briefly, TMA tissue sections were deparaffinized and rehydrated with EZprep concentrate (10×) solution (Ventana Medical Systems). Antigen retrieval was performed with cell conditioning 1 (Ventana Medical Systems) at 95°C for 30 min. Slides were then treated with iView DAB (3,3'-diaminobenzidine tetrahydrochloride) Inhibitor (Ventana Medical Systems) and incubated with 100 µL primary antibody (1∶100) for 90 min. The following primary antibodies were used for IHC staining: pAMPK (Thr 172), pACC (Ser 79), and phosphorylated extracellular signal-regulated kinase (pErk) (Thr 202/Tyr 204) (All Cell Signaling Technology; Danvers, MA, USA) and immunoglobulin G (clone SP3) (Thermo Fisher Scientific, USA). These antibodies were chosen because they have been investigated in other studies [15]–[17]. Bound antibody was detected using the iView DAB Detection Kit (Ventana Medical Systems) according to the manufacturer’s instructions.

### Scoring of pAMPK, pACC, and pErk IHC Expression in TMAs

**Figure 1:**
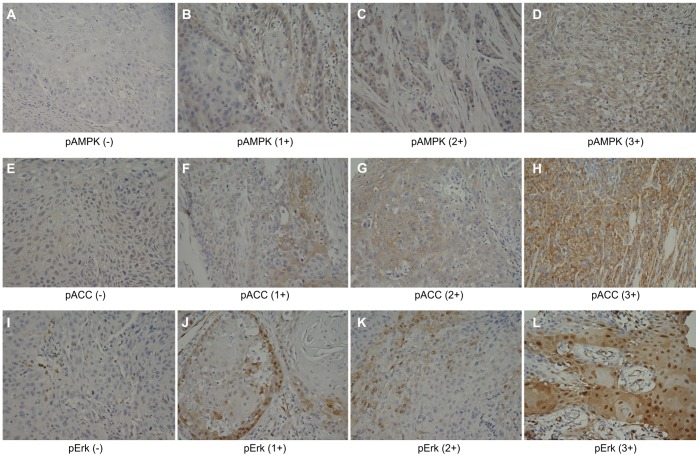
Representative immunohistochemistry scores for pAMPK, pACC, and pErk in SCCHN specimens. pAMPK and pACC are predominantly expressed in the cytoplasm of tumor cells: (A-D) pAMPK staining (0−3+) and (E-H) pACC staining (0−3+) (× 200 magnification. Expression of pErk is both cytoplasmic and nuclear: (I-L) pErk staining (0−3+) (×200 magnification).

For pAMPK and pACC, cytoplasmic staining was scored because little to no nuclear staining was observed. Combined nuclear and cytoplasmic staining was used to score pErk IHC expression. The expression of these phosphorylated proteins was reviewed by 2 pathologists (Y-H. L. and C-L. C.) and quantified as previously described [15]. A composite score was generated from the staining intensity (0, 1+, 2+, and 3+) and the percentage of the extent of reactivity. Scores were averaged over replicate cores to generate a final IHC score for each tumor. Representative examples of each score are presented in Figure 1. Tumors with IHC score values ≥1+ were considered positive and those with a score of 0 were considered negative. A pACC IHC score value of 1+ or 2+ was considered low expression, and a pACC IHC score value of 3+ was considered high expression.

### Statistical Analysis

Statistical analysis was performed to determine the association of pAMPK expression with clinicopathological features and pACC and pErk expression. Categorical variables were compared using χ^2^ or Fisher’s exact test, and correlations were assessed using Spearman’s rank correlation test. OS was defined as the time from the date of first treatment until the date of death. The OS rates according to pAMPK, pACC, and pErk expression were estimated using the Kaplan–Meier method and compared using the log-rank test. Univariate analysis was used to test the association between the expression of each phosphoprotein and survival. Significant associations were further tested in the multivariate analysis using Cox proportional hazards model adjusted for other covariates including age, gender, tumor/nodal status, stage, primary site, and adjuvant treatment. All statistical tests were 2-sided, and significance was defined as a *P* value <0.05.

## Results

### Expression of pAMPK and pACC in TMAs

Consistent with the Taiwan National Health Insurance Research Database [18], our SCHHN patients were predominantly male (92.4%) (Table 1). pAMPK and pACC were found mainly in the cytoplasm of primary human tumor specimens, while normal mucosa tissues were negative or weakly stained in the nuclei (Fig. S1). The distribution and average intensity of pAMPK and pACC staining were different between the normal and cancer tissues. The positive rates of pAMPK and pACC expression in 118 SCHNN cases were 64.4% (76/118) and 68.6% (81/118), respectively. Twenty-three of 118 patients (19.5%) exhibited high expression of pACC (3+).

### Association of pAMPK Expression with Clinicopathological Features

The association of pAMPK expression with clinicopathological features is shown in Table 2. pAMPK expression was not significantly associated with gender, nodal status, stage, or (neo)adjuvant treatment. In contrast, pAMPK expression was significantly correlated with age, T stage, and tumor site. The number of patients age <60 years (75.0% vs. 54.8%; *P = *0.024; χ^2^ test) and those with early-stage (T1/T2) tumor status (77.6% vs. 57.1%; *P* = 0.020; χ^2^ test) and oral cavity tumors (94.7% vs. 81.0%; *P* = 0.026; Fisher’s exact test) was higher in the positive pAMPK expression group than in the negative pAMPK expression group. Given that chemotherapy and radiotherapy can activate AMPK [19], expression levels of pAMPK in SCCHN patients were re-examined after excluding those who had received radiotherapy or chemotherapy prior to tumor specimen collection. In this subpopulation (*n = *102), the number of patients age <60 years (78.8% vs. 52.8%; *P* = 0.006; χ^2^ test) and those with early-stage (T1/T2) tumor status (84.9% vs. 61.1%; *P* = 0.007; χ^2^ test) was higher in the positive pAMPK expression group than in the negative pAMPK expression group (Table S1). Although not statistically significant, a trend of increased pAMPK expression in SCCHN patients with the oral cavity as the primary tumor site was observed (95.5% for positive pAMPK expression vs. 83.3% for negative pAMPK expression; *P* = 0.064; Fisher’s exact test).

**Table 2:** Association of pAMPK expression with clinicopathological features and pACC and pERK expression.

	Negative pAMPK	Positive pAMPK	*P* value
	*n = *42 (%)	*n* = 76 (%)	
**Age, median (range)**			
<60 years	23 (54.8)	57 (75.0)	0.024*^C^
≥60 years	19 (45.2)	19 (25.0)	
**Gender**			
Female	3 (7.1)	6 (7.9)	1.000^F^
Male	39 (92.9)	70 (92.1)	
**(Neo)Adjuvant**			
None	20 (47.6)	37 (48.7)	0.912^C^
Any	22 (52.4)	39 (51.3)	
**T**			
T1/T2	24 (57.1)	59 (77.6)	0.020*^C^
T3/T4	18 (42.9)	17 (22.4)	
**N**			
N0	31 (73.8)	50 (65.8)	0.369^C^
N1/N2	11 (26.2)	26 (34.2)	
**Stage**			
I/II	21 (50.0)	42 (55.3)	0.583^C^
III/IV	21 (50.0)	34 (44.7)	
**Site**			
Non-oral cavity	8 (19.0)	4 (5.3)	0.026*^F^
Oral cavity	34 (81.0)	72 (94.7)	
**Differentiation**			
Well to moderate	34 (81.0)	53 (69.7)	0.200^F^
Poorly	8 (19.0)	23 (30.3)	
**Margin**			
Negative	27 (67.5)	58 (77.3)	0.253^C^
Positive	13 (32.5)	17 (22.7)	
**Lymphovascular invasion**			
Negative	25 (78.1)	51 (83.6)	0.577^F^
Positive	7 (21.9)	10 (16.4)	
**pACC**			
Negative or low expression	33 (78.6)	62 (81.6)	0.809^F^
High expression	9 (21.4)	14 (18.4)	
**pErk**			
Negative	33 (78.6)	50 (65.8)	0.206^F^
Positive	9 (21.4)	26 (34.2)	

C χ^2^ test; ^F^Fisher’s exact test; *P<0.05.

T, tumor; N, node; pACC, phosphorylated acetyl-CoA carboxylase; pErK, phosphorylated extracellular signal-regulated kinase; pAMPK, phosphorylated AMP-activated protein kinase; *n*, patient number.

### Association of pAMPK with pACC and pErk

Because ACC is a substrate of AMPK, the correlation between pAMPK and pACC expression was examined. pAMPK and pACC expression were not significantly associated after adjusting for T stage, tumor stage, and primary tumor site (data not shown). Of 76 patients with positive pAMPK expression, 13 were scored as pAMPK 3+. However, only 2 of these 13 patients had high pACC expression. Among patients with negative pAMPK expression (*n = *42), 9 patients had high pACC expression (Table 2). These findings suggested that phosphorylation of ACC in SCCHN may be AMPK independent. However, in the subgroup analysis according to nodal status, pAMPK and pACC expression were significantly correlated in node-positive, but not node-negative, disease (*P = *0.028; Fisher’s exact test) (Table S2).

High levels of epidermal growth factor receptor (EGFR) and its downstream target Erk have been correlated with poor prognosis in SCCHN [20]. Therefore, we also determined the association of pAMPK with pErk expression. pAMPK expression was not significantly associated with pErk expression (*P* = 0.206; Fisher’s exact test) (Table 2).

### Association of pAMPK, pACC, and pErk with Survival

**Figure 2:**
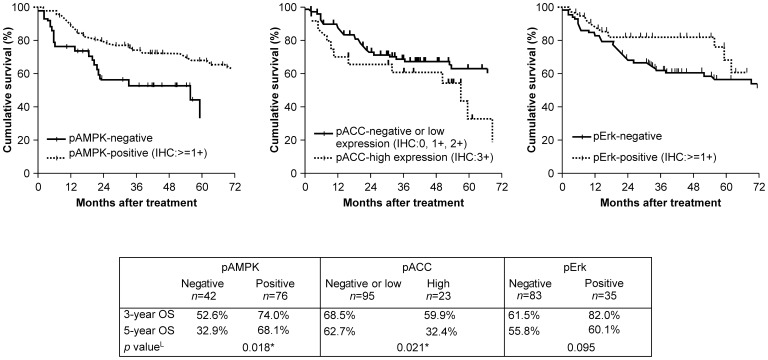
Kaplan–Meier survival curves for pAMPK, pACC, and pErk expression groups. Survival curves for (A) pAMPK, (B) pACC, and (C) pErk expression in patient groups compared using the log-rank test.

Kaplan–Meier survival curves for the pAMPK, pACC, and pErk expression groups are shown in Figure 2. Survival was significantly different between the 2 AMPK expression groups (*P = *0.018; log-rank test). In the univariate analysis, the mean OS of AMPK-positive patients was 6.5 years (standard error [SE]: 0.4 years), and the 3 and 5-year survival rates were 74.0% and 68.1%, respectively. Among AMPK-negative patients, the mean OS was 3.1 years (SE: 0.3 years), and the 3 and 5-year survival rates were 52.6% and 32.9%, respectively. Survival was also significantly different between the 2 pACC expression groups (*P = *0.021; log-rank test). The mean OS of patients with high pACC expression was 4.1 years (SE: 0.7 years), and the 3 and 5-year survival rates were 59.9% and 32.4%, respectively. The mean OS of patients with negative or low pACC expression was 6.3 years (SE: 0.4 years), and the 3 and 5-year survival rates were 68.5% and 62.7%, respectively. pErk expression did not reach statistical significance in the survival analysis.

Multivariate analysis using Cox proportional hazards models was performed to determine the prognostic significance of the clinicopathological variables, pAMPK, pACC, and pErk. Age ≥60 years, advanced (T3/T4) tumor stage, positive nodal status, and high pACC expression were independently associated with worse OS after adjusting for covariates (adjusted hazard ratios [HRs]: 2.97, 3.39, 2.54, and 2.48, respectively) (Table 3). Because positive pAMPK expression was associated with early-stage (T1/T2) tumor status and oral cavity tumors, we examined the prognostic significance of pAMPK in this patient subgroup (data not shown). In the subgroup multivariate analysis, pAMPK expression was not associated with OS (adjusted HR: 0.44; 95% CI: 0.16–1.25; *P = *0.124), whereas high pACC expression had a significantly higher adjusted HR of 3.42 (95% CI: 1.19–9.83; *P = *0.023).

**Table 3:** Multivariate analysis of overall survival.

	Adjusted HR (95% CI)	*P* value
**Age**		
≥60 vs. <60 years	2.97 (1.54–5.72)	0.001*
**Gender**		
Male vs. Female	0.49 (0.18–1.31)	0.153
**Site**		
Oral cavity vs. Non-oral cavity	1.50 (0.60–3.78)	0.390
**T**		
T3/T4 vs. T1/T2	3.39 (1.75–6.57)	<0.001*
**N**		
Positive vs. Negative	2.54 (1.22–5.27)	0.012*
**(Neo)Adjuvant**		
Any vs. none	1.16 (0.57–2.36)	0.683
**pAMPK**		
Positive (≥1+) vs. Negative	0.66 (0.35–1.23)	0.188
**pACC**		
High vs. Negative or low expression	2.48 (1.17–5.28)	0.018*
**pErK**		
Positive (≥1+) vs. negative	0.59 (0.26–1.35)	0.211

*P<0.05. T, tumor; N, node; pAMPK, phosphorylated AMP-activated protein kinase; pACC, phosphorylated acetyl-CoA carboxylase; pErK, phosphorylated extracellular signal-regulated kinase; HR, hazard ratio; 95% CI, 95% confidence interval.

To further evaluate the prognostic value of high pACC expression and pAMPK expression, subgroups analyses according to nodal status and clinical stage were performed. Multivariate analysis was performed in SCCHN patients with node-negative (N0) and node-positive (N1 and N2) disease. Among node-negative SCCHN patients, only those with more advanced (T3/T4) tumor status had worse outcomes (adjusted HR: 3.70; 95% CI: 1.43–9.58) (Table 4). Elevated expression of pACC did not affect OS. Although statistical significance was not reached, pAMPK expression showed a trend for correlation with better outcome (adjusted HR: 0.45; 95% CI: 0.19–1.08). In SCCHN patients with node-positive disease, high pACC expression (adjusted HR: 17.58; 95% CI: 3.50–88.18) and age ≥60 years (adjusted HR: 5.25; 95% CI: 1.68–16.36) was significantly associated with patient survival. Advanced (T3/T4) tumor status, (neo)adjuvant treatment, and expression of pAMPK and pErk did not impact OS. Similar results were observed in the subgroup analysis according to clinical stage. Age ≥60 years and high pACC expression consistently showed a significant impact on OS in stage III and stage IV patients, with adjusted HRs of 3.36 (95% CI: 1.34–8.46) and 2.82 (95% CI: 1.06–7.48), respectively. Expression of pAMPK and pErk did not affect OS regardless of primary tumor site, tumor status, or clinical stage.

**Table 4:** Multivariate analysis of overall survival according to nodal status.

	Node negative (*n* = 81)		Node positive (*n* = 37)	
Variables	Adjusted HR (95% CI)	*P* value	Adjusted HR (95% CI)	*P* value
**Age**				
≥60 vs. <60 years	2.26 (0.89–5.70)	0.085**	5.25 (1.68–16.36)	0.004*
**Gender**				
Male vs. Female	0.21 (0.05–0.81)	0.024*	0.74 (0.15–3.65)	0.713
**Site**				
Oral cavity vs. Non-oral cavity	0.93 (0.29–2.96)	0.898	2.39 (0.40–14.28)	0.339
**T**				
T3/T4 vs. T1/T2	3.70 (1.43–9.58)	0.007*	2.17 (0.69–6.86)	0.186
**(Neo)Adjuvant treatment**				
Any vs. None	1.14 (0.46–2.86)	0.774	1.54 (0.27–8.74)	0.625
**pAMPK**				
Positive vs. negative	0.45 (0.19–1.08)	0.072**	0.67 (0.23–1.97 )	0.472
**pACC**				
High expression vs. negative or low expression	1.55 (0.61–3.88)	0.355	17.58 (3.50–88.18)	<0.001*
**pErk**				
Positive vs. negative	0.49 (0.15–1.58)	0.232	0.41 (0.11–1.53)	0.184

*P<0.05; **P<0.1.

T, tumor; pAMPK, phosphorylated AMP-activated protein kinase; pACC, phosphorylated acetyl-CoA carboxylase; pErK, phosphorylated extracellular signal-regulated kinase; *n*, patient number; HR, hazard ratio; 95% CI, 95% confidence interval.

## Discussion

In the present study, we determined the expression levels of pAMPK and pACC in surgical tumor specimens from 118 patients with SCCHN using high-throughput tissue microarrays and evaluated their impact on survival. The antibodies used in the study have been investigated and validated by other researchers [15]–[17]. We observed that pAMPK expression was significantly increased in younger patients (age <60 years), early-stage (T1/T2) tumor status, and tumors originating from the oral cavity. In the multivariate analysis, expression of pAMPK was not significantly correlated with patient survival. In addition to age ≥60 years and advanced (T3/T4) tumor status, we identified high expression of pACC (3+) as an independent prognostic marker in SCCHN patients with node-positive or advanced (III/IV)-stage disease, a finding that has not been previously reported.

pAMPK expression was associated with increased survival in lung cancer patients, particularly in those with adenocarcinoma, indicating that pAMPK expression may be useful as a prognostic marker for lung cancer [15]. In the current study, pAMPK expression was observed more often in patients with early-stage (T1/T2) tumor status than in those with advanced (T3/T4) tumor status, suggesting a loss of protection from the pAMPK pathway with disease progression. Although univariate analysis showed a trend of better survival in all SCCHN patients with positive pAMPK expression, pAMPK did not have an impact on OS in the multivariate analysis after adjusting for tumor status, age, tumor site, and other factors.

ACC is an important downstream target of pAMPK. ACC is a key regulatory enzyme in fatty acid *de novo* biosynthesis and lipogenesis [21]. ACC catalyzes the carboxylation of acetyl-CoA to produce malonyl-CoA, an intermediate of fatty acid synthesis. This activity of ACC is inhibited by its phosphorylation. In humans, there are 2 ACC isoforms: ACC1, which is mainly located in the cytosol, and ACC2, which is located mainly in mitochondria [22]. ACC1 is expressed in all cell types but is enriched in lipogenic tissues [23]. Knockout mice with homozygous mutant ACC1^?/?^ are embryonic lethal, whereas ACC2^?/?^ mice live well and gain less weight than their wild-type counterparts [24]. Inhibition of ACC has been shown to have antitumor activity in various cancers. Several studies have shown that dephosphorylation of ACC1 at Ser 79 is prevented by its interaction with breast cancer protein 1 (BRCA1) [25]–[27], a DNA repair gene involved in maintaining genetic stability. This finding suggested that inactivation of ACC1 may be involved in BRCA1-mediated tumor suppression. Inhibition of ACC induced apoptosis in breast and prostate cancer cells [28]–[30], suggesting ACC is essential for cancer cell survival [31]. In contrast to previous *in?vitro* and *in?vivo* studies, our data indicated that inhibition of ACC did not have a protective effect in SCCHN patients. To assess the prognostic value of ACC inhibition in our study, we used a specific antibody to detect the phosphorylation of ACC1 at Ser 79, the major phosphorylation site of AMPK [24]. We identified elevated expression of pACC as an independent prognostic marker for SCCHN, particularly in patients with node-positive and advanced (III/IV)-stage disease. The discrepancy between our study and the previous studies may indicate a gap in the present knowledge regarding the energy metabolism of cancer cells among different experimental systems (*in?vitro*, *in?vivo*, or patient samples). Further evaluation of the crosstalk between metabolic and cell survival signals and its role in cancer development and progression is warranted.

The classical target of AMPK is ACC. In the present study, pACC expression was not significantly associated with pAMPK expression after adjusting for T stage, tumor stage, and primary tumor site (data not shown). However, in the subgroup analysis, we observed a statistically significant correlation between positive expression of pAMPK and pACC in node-positive disease. This correlation was not observed in SCCHN patients with node-negative (N0) disease. These findings suggested that AMPK-dependent and AMPK-independent pathways may inactivate ACC in SCCHN. In addition to AMPK, ACC is also phosphorylated by many other kinases, including protein kinase C (PKC) and casein kinase 2 (CK2) [32]. Overexpression of PKC and CK2 has been reported in SCCHN tumors and are associated with aggressive clinical behavior and worse prognosis [33]–[35]. Further studies are needed to investigate other phosphoregulators of ACC1 and their role in SCCHN tumor progression.

Although overexpression of EGFR has been correlated with poor prognosis in SCCHN, few SCCHN patients respond to EGFR-targeted drugs [36]. In the present study, expression of pErk, an important downstream target of EGFR, did not significantly affect patient survival in the multivariate analysis, suggesting that SCCHN may not be associated with this pathway. Therefore, new therapeutic strategies are needed to treat this disease.

## Conclusions

Our study is the first to demonstrate pAMPK and pACC expression in SCCHN and their impact on survival. Although pAMPK expression was found to be higher in patients with younger age, early-stage tumor status, and oral cavity tumors, it was not an independent factor for predicting patient outcome in the multivariate analysis. We identified high expression of pACC as a worse prognostic factor, particularly in patients with node-positive or stage III/IV disease. Given that lymph node metastasis in SCCHN is a major prognostic factor, our findings suggest a distinct cellular signaling pathway in this group of patients. Our results suggested that pACC may play a role in tumor progression of SCCHN and may help to identify patient subgroups at high risk for poor disease outcome. Future studies are required to validate pACC as a therapeutic target for SCCHN.

## Supporting Information

Figure S1 **Representative examples of SCCHN tumor section and normal mucosal tissue stained by pAMPK and pACC antibodies.**
(TIF)

Table S1 **Association of pAMPK expression with clinicopathological features in patients not receiving neoadjuvant chemotherapy or radiotherapy.**
(DOC)

Table S2 **Correlation between pAMPK and pACC in node-negative (N0) and node-positive (N1/N2) patients.**
(DOC)
