# Supplementary figures and images for: Association between Phosphorylated AMP-Activated Protein Kinase and Acetyl-CoA Carboxylase Expression and Outcome in Patients with Squamous Cell Carcinoma of the Head and Neck
Source: PLoS One. 2014 Apr 25;9(4):e96183. doi: 10.1371/journal.pone.0096183 (PMC4000216; doi:10.1371/journal.pone.0096183)

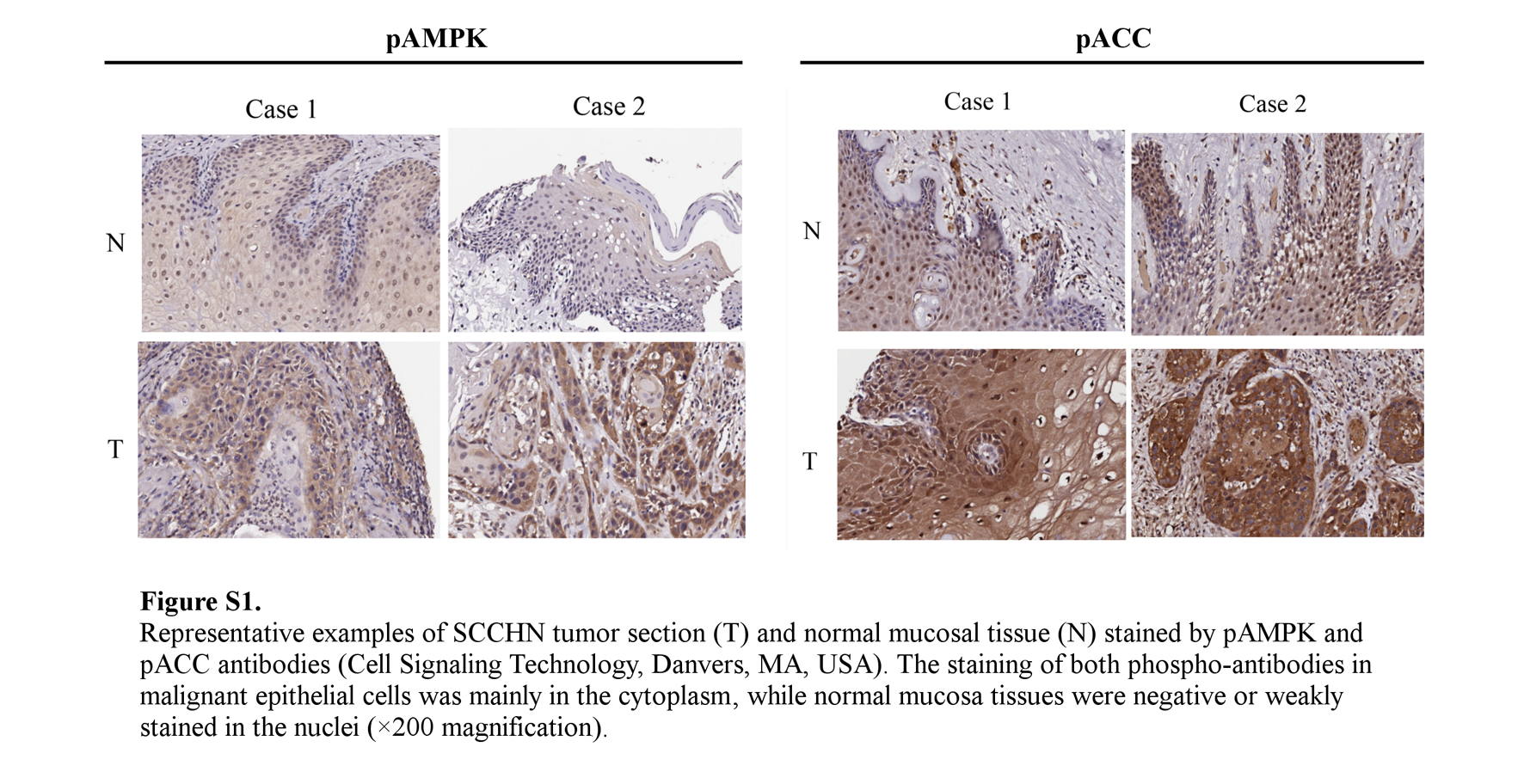

Supplement: Figure S1 — Representative examples of SCCHN tumor section and normal mucosal tissue stained by pAMPK and pACC antibodies. (TIF) [file pone.0096183.s001.tif]
