# Supplementary material for: Association between Phosphorylated AMP-Activated Protein Kinase and Acetyl-CoA Carboxylase Expression and Outcome in Patients with Squamous Cell Carcinoma of the Head and Neck
Source: PLoS One. 2014 Apr 25;9(4):e96183. doi: 10.1371/journal.pone.0096183 (PMC4000216; doi:10.1371/journal.pone.0096183)
Supplement: Table S1 — Association of pAMPK expression with clinicopathological features in patients not receiving neoadjuvant chemotherapy or radiotherapy. (DOC) [file pone.0096183.s002.doc]

**Table S1 Association of pAMPK expression with clinicopathological features in patients not receiving** neoadjuvant chemotherapy or radiotherapy

|  | **Negative pAMPK**  *n* = 36 (%) | **Positive pAMP**K  *n* = 66 (%) | ***P* value** |
| --- | --- | --- | --- |
| **Age** |  |  |  |
| < 60 years | 19 (52.8) | 52 (78.8) | 0.006*^C^ |
| ≥ 60 years | 17 (47.2) | 14 (21.2) |  |
| **Gender** |  |  |  |
| Female | 3 (8.3) | 6 (9.1) | 1.000^F^ |
| Male | 33 (91.7) | 60 (90.9) |  |
| **T** |  |  |  |
| T1/T2 | 22 (61.1) | 56 (84.9) | 0.007*^C^ |
| T3/T4 | 14 (38.9) | 10 (15.1) |  |
| **N** |  |  |  |
| N0 | 28 (77.8) | 45 (68.2) | 0.363^F^ |
| N1/N2 | 8 (22.2) | 21 (31.8) |  |
| **Stage** |  |  |  |
| I/II | 19 (52.8) | 40 (60.6) | 0.444^C^ |
| III/IV | 17 (47.2) | 26 (39.4) |  |
| **Site** |  |  |  |
| Non-oral cavity | 6 (16.7) | 3 (4.5) | 0.064^F^ |
| Oral cavity | 30 (83.3) | 63 (95.5) |  |
| **Differentiation** |  |  |  |
| Well to moderate | 28 (77.8) | 47 (71.2) | 0.639^F^ |
| Poor | 8 (22.2) | 19 (28.8) |  |
| **Margin** |  |  |  |
| Negative | 25 (73.5) | 54 (83.1) | 0.298^F^ |
| Positive | 9 (26.5) | 11 (16.9) |  |
| **Lymphovascular invasion** |  |  |  |
| Negative | 23 (82.1) | 45 (84.9) | 0.758^F^ |
| Positive | 5 (17.9) | 8 (15.1) |  |
| **pACC** |  |  |  |
| Negative or low expression | 29 (80.6) | 55 (83.3) | 0.788^F^ |
| High expression (3+) | 7 (19.4) | 11 (16.7) |  |
| **pErK** |  |  |  |
| Negative | 28 (77.8) | 43 (65.2) | 0.260^F^ |
| Positive (≥1+) | 8 (22.2) | 23 (34.8) |  |

^C^χ^2^ test; ^F^Fisher’s exact test; **P*<0.05.

T, tumor; N, node; pACC, phosphorylated acetyl-CoA carboxylase; pErK, phosphorylated extracellular signal-regulated kinase; pAMPK, phosphorylated AMP-activated protein kinase; *n*, patient number.
