# Supplementary material for: Association between Phosphorylated AMP-Activated Protein Kinase and Acetyl-CoA Carboxylase Expression and Outcome in Patients with Squamous Cell Carcinoma of the Head and Neck
Source: PLoS One. 2014 Apr 25;9(4):e96183. doi: 10.1371/journal.pone.0096183 (PMC4000216; doi:10.1371/journal.pone.0096183)
Supplement: Table S2 — Correlation between pAMPK and pACC in node-negative (N0) and node-positive (N1/N2) patients. (DOC) [file pone.0096183.s003.doc]

**Table S2 Correlation between pAMPK and pACC in node-negative (N0) and node-positive (N1/N2) patients**

|  | **Node negative pAMPK (*n* = 81)** | | | **Node positive pAMPK (*n* = 37)** | | |
| --- | --- | --- | --- | --- | --- | --- |
|  | **Negative** | **Positive** | **P** | **Negative** | **Positive** | **P** |
| **pACC** |  |  |  |  |  |  |
| Negative | 7 (22.6%) | 17 (34.0%) | 0.32 ^F^ | 7 (63.6%) | 6 (23.1%) | 0.03* ^F^ |
| Positive | 24 (77.4%) | 33 (66.0%) |  | 4 (36.4%) | 20 (76.9%) |  |
| **pACC** |  |  |  |  |  |  |
| Negative or low expression | 24 (77.4%) | 39 (78.0%) | 1.00^F^ | 9 (81.8%) | 23 (88.5%) | 0.62^F^ |
| High expression (3+) | 7 (22.6%) | 11 (22.0%) |  | 2 (18.2%) | 3 (11.5%) |  |
| **pACC** |  |  |  |  |  |  |
| Spearman | -0.0325 |  | 0.773 | 0.3260 |  | 0.049* |

^F^Fisher’s exact test; **P*<0.05.

pACC, phosphorylated acetyl-CoA carboxylase; *n*, patient number; pAMPK, phosphorylated AMP-activated protein kinase.
